# Matters of the Heart

**Published:** 2008-03-15

**Authors:** Lynne S. Wilcox

The science fiction film *Fantastic Voyage* was released in the United States in 1966. Its plotline, preceding nanotechnology by many years, begins with a scientist who needs emergency medical treatment for a stroke. However, this surgery can only be accomplished from inside his body. Intrepid medical personnel enter a submarine, which becomes miniaturized and is injected into a blood vessel. The submarine dodges red blood cells and macrophages as it sweeps through the heart, and the crew exits the submarine and is tossed about in the winds of the lungs. The special effects were among the most creative (and psychedelic) of the time, winning the film two Academy Awards. The voyage through the body took viewers into an intimate adventure where biologic reactions controlled all events. Though the science was sometimes flawed, the models of the interior of a blood vessel were stunning. One could hardly find a better documented microscopic view of the cardiovascular system.

But now, put away that microscope. Stand at the patient's bedside and speak with his family. Step back and pause in the hospital emergency room; travel in the emergency response vehicle. Helicopter above the city and observe the worksites and schools. At higher elevations, look for the neighborhoods and the urban centers. Fly across the country and find the rural communities and the ethnic enclaves; circle the state and national legislative houses. In the United States in 2000 more than 711,000 deaths from heart attack and 168,000 deaths associated with stroke occurred, representing 37% of all deaths in the country (1). If we are to protect people from heart attack and stroke, we must move beyond intravascular space to understand the cultures, behaviors, and systems that contribute to the risk factors that cause cardiovascular disease.

This issue of *Preventing Chronic Disease* explores how public health programs can be used in conjunction with the efforts of private and public groups to reduce the morbidity and mortality of cardiovascular disease. We thank Ms Mirium Patanian of the Cardiovascular Health Council, Washington State Department of Health, and Dr Nell Brownstein of the Division for Heart Disease and Stroke Prevention, National Center for Chronic Disease Prevention and Health Promotion, Centers for Disease Control and Prevention, for serving as guest editors for this issue. Ms Patanian and Dr Brownstein discuss the issue's articles in depth (2,3), setting forth the challenges of addressing these complex diseases and reiterating our recognition that multiple contextual factors affect their prevalence.

We are on our own fantastic voyage. Since the mid–20th century we have learned methods — including decreasing tobacco use, controlling high blood pressure, reducing dietary fat, and improving clinical care — for reducing the mortality and morbidity associated with cardiovascular disease. Between 1950 and 2004, cardiovascular disease death rates dropped from 587 to 217 per 100,000 age-adjusted population (5), and stroke deaths dropped from 181 to 50 (4).

We have projected a virtual tour of the country, because the next advances in heart attack and stroke reduction will depend not only on individual behaviors and medical care but also on population-based interventions (5). We must better understand the reasons for racial and ethnic differences in disease and death rates, explore opportunities to reach underserved populations, and develop policy and environmental strategies for protecting people at risk. We will continue to explore newly recognized factors that may affect disease prevalence, such as genetics and infectious agents. National surveillance of morbidity and mortality related to cardiovascular disease and community-level interventions are needed. We will design better secondary interventions to reduce disability and improve quality of life for people with existing cardiovascular conditions.

*Fantastic Voyage* ends as the crew of five, minus the evil scientist, saves one man's life and escapes through a tear duct. For heart attack and stroke, the health of millions of people is at stake, and we do not have the luxury of five health professionals for each person at risk. No psychedelic adventures appear to be in store for us; it will require more than science fiction submarines and a handful of heroes to continue improving health. But based on our history in combating these events, the future looks promising.
